# Genome-wide profiling of histone H3K4me3 and H3K27me3 modifications in individual blastocysts by CUT&Tag without a solid support (NON-TiE-UP CUT&Tag)

**DOI:** 10.1038/s41598-022-15417-x

**Published:** 2022-07-11

**Authors:** Kazuki Susami, Shuntaro Ikeda, Yoichiro Hoshino, Shinnosuke Honda, Naojiro Minami

**Affiliations:** Laboratory of Reproductive Biology, Graduate School of Agriculture, Kyoto University, Kyoto, 606-8502 Japan

**Keywords:** Epigenomics, Embryology

## Abstract

Individual analysis of the epigenome of preimplantation embryos is useful for characterizing each embryo and for investigating the effects of environmental factors on their epigenome. However, it is difficult to analyze genome-wide epigenetic modifications, especially histone modifications, in a large number of single embryos due to the small number of cells and the complexity of the analysis methods. To solve this problem, we further modified the CUT&Tag method, which can analyze histone modifications in a small number of cells, such that the embryo is handled as a cell mass in the reaction solutions in the absence of the solid-phase magnetic beads that are used for antibody and enzyme reactions in the conventional method (NON-TiE-UP CUT&Tag; NTU-CAT). By using bovine blastocysts as a model, we showed that genome-wide profiles of representative histone modifications, H3K4me3 and H3K27me3, could be obtained by NTU-CAT that are in overall agreement with the conventional chromatin immunoprecipitation-sequencing (ChIP-seq) method, even from single embryos. However, this new approach has limitations that require attention, including false positive and negative peaks and lower resolution for broad modifications. Despite these limitations, we consider NTU-CAT a promising replacement for ChIP-seq with the great advantage of being able to analyze individual embryos.

## Introduction

A close investigation of the epigenome of early preimplantation embryos is important for validating the temporal and spatial regulation of gene expression that is crucial for their development and examining the impact of environmental factors on their epigenome. Post-translational histone modifications are representative epigenetic modifications, and genome-wide investigations of these modifications in early embryos have been performed using mainly the chromatin immunoprecipitation-sequencing (ChIP-seq) method^1–3^. However, because early embryos are composed of a very small number of cells (~ 100 cells), it is difficult to analyze a single embryo. However, several methods have been proposed recently to overcome the disadvantages of ChIP-seq, which requires a large number of cells for analysis, and these methods have been applied to the analysis of single or small numbers of preimplantation embryos^4,5^. First, Henikoff and colleagues, based on Laemmli’s Chromatin ImmunoCleavage (ChIC) strategy^6^, proposed the Cleavage Under Targets and Release Using Nuclease (CUT&RUN)^7,8^. Although CUT&RUN can produce high quality data from as few as 100–1000 cells, it has disadvantages in terms of the time, cost, and effort required for purification, end polishing, and adapter ligation of micrococcal nuclease (MNase)-cleaved DNA fragments that are released into the supernatant^7,8^. However, they overcame these disadvantages and developed a new method called Cleavage Under Targets and Tagmentation (CUT&Tag), which uses a fusion protein of Tn5 transposase and protein A (pA-Tn5), instead of pA-MNase, loaded with sequencing adapters to ligate the adapters directly to the cleaved fragments^9,10^. This strategy results in in situ formation of PCR-amplifiable adapter-ligated fragments for sequencing, unlike in the case of CUT&RUN. Therefore, we expected that CUT&Tag could be performed without binding cells or isolated cell nuclei to the solid phase, as in conventional CUT&RUN and CUT&Tag. Here, we report profiling of representative histone modifications, H3K4me3 and H3K27me3, in single bovine blastocysts as a model using CUT&Tag without a solid phase (NON-TiE-UP CUT&Tag; NTU-CAT).

## Results

### Schema for NTU-CAT

**Figure 1:**
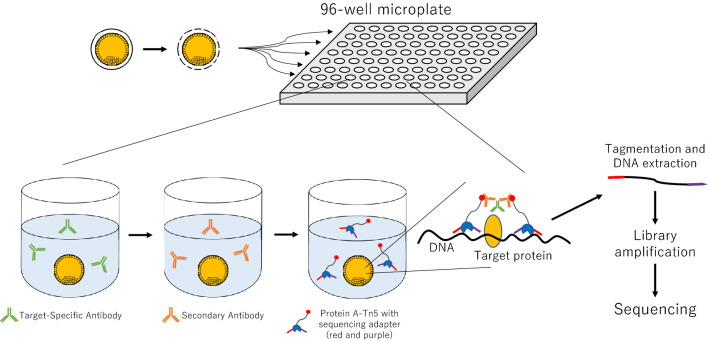
Schematic diagram of NON-TiE-UP CUT&Tag (NTU-CAT). See the “Methods” section for details.

The schema for NTU-CAT is shown in Fig. 1. After removal of the zona pellucida, the embryos were immersed in a primary antibody solution containing a detergent (digitonin) and incubated without binding of the cells or isolated nuclei to a solid phase (concanavalin A-coated magnetic beads) as in the conventional method. Subsequent secondary antibody reactions, tethering of pA-Tn5 fusion protein with sequencing adapters, and tagmentation were also performed without a solid support, by transferring the embryos to the respective reaction solutions. The tagmented DNA was extracted and PCR amplified using index primers, and the library after purification was used for sequencing. The numbers of sequencing reads generated and processed are summarized in Table S1.

### H3K4me3 profile of blastocysts assessed by NTU-CAT

**Figure 2:**
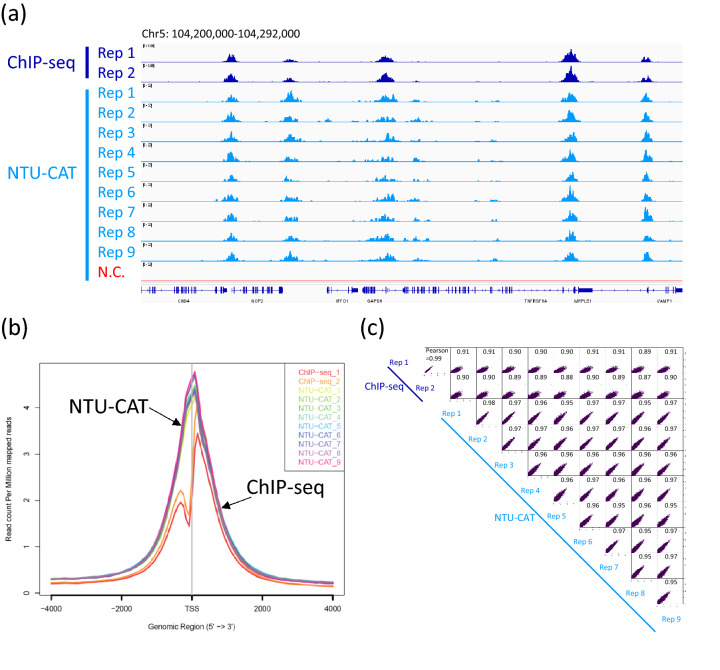
Comparison of NTU-CAT and ChIP-seq results for H3K4me3 in bovine blastocysts. (**a**) Snapshot of the H3K4me3 landscape in a 92-kb region (chromosome 5) that includes the *GAPDH* TSS. The NTU-CAT-derived peaks in nine replicates were visualized using Integrative Genomics Viewer^29^ alongside the peaks from our previous ChIP-seq analysis of a cohort of blastocysts^11^. N.C. is the negative control in which the primary antibody was omitted from the NTU-CAT procedure. (**b**) Average profile plot of the H3K4me3 signal around genome-wide TSSs. The signals from the nine replicates of NTU-CAT and duplicates of ChIP-seq^11^ are shown. The average profile plots were generated by ngs.plot^28^. (**c**) Correlation analysis of duplicates of ChIP-seq and nine replicates of NTU-CAT for H3K4me3 in bovine blastocysts. Scatterplots of these pairwise comparisons are shown with Pearson correlation coefficients. Bin sizes of 10 kb and natural log transformation after adding 1 were used for drawing with deepTools (https://deeptools.readthedocs.io/en/develop/).

Among the various types of histone methylation, H3K4me3 is a typical transcription-promoting marker that generally shows sharp and distinct modifications^1–3^. Therefore, we initially targeted this modification for NTU-CAT application. Because NTU-CAT allows for rapid analysis of histone modifications from individual embryos, we could readily obtain data from multiple embryos. A snapshot of the NTU-CAT peaks from nine replicates (i.e., nine single blastocysts) is shown in Fig. 2a, alongside the peaks from our previous ChIP-seq analysis of a cohort (n =  ~ 11) of blastocysts for H3K4me3 modification^11^. The overall landscape depicted by the location and shape of the peaks was very similar between the two methods, except for subtle differences in the shape of the peaks (Fig. 2a). Figure 2b shows the average profile plots of the H3K4me3 signal around the transcript start site (TSS) regions in these experiments. A striking difference between the NTU-CAT and ChIP-seq profiles was that the valley-like shapes near TSSs detected in ChIP-seq were not detected in NTU-CAT, which is in line with other studies on CUT&Tag^9,12^. However, the peak shape of NTU-CAT could be approximated to that of ChIP-seq. Supportively, pairwise comparisons of H3K4me3 signals showed a high correlation between the methods and replicates (Fig. 2c).

**Figure 3:**
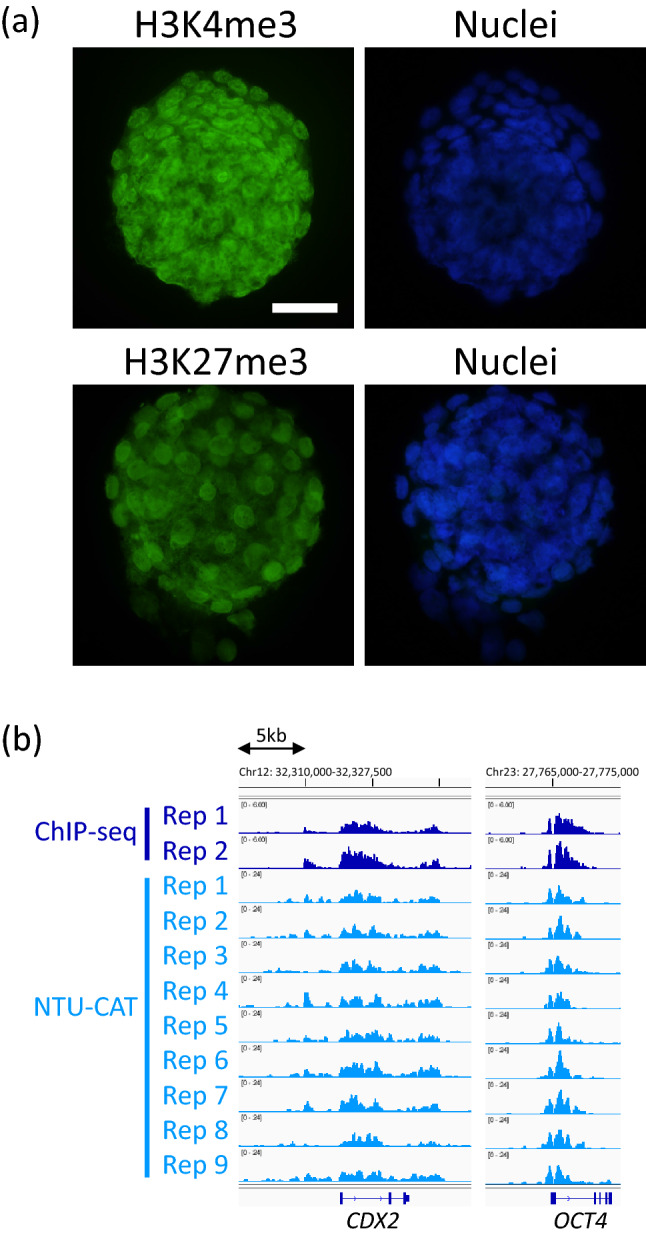
Permeability of antibodies in NTU-CAT. (**a**) Immunofluorescent images of H3K4me3 (upper, left) and H3K27me3 (lower, left) assessed by replacing the secondary antibody with a fluorochrome-conjugated antibody are shown alongside the nuclear counter-staining by Hoechst 33342 (right). Scale bar represents 50 µm. (**b**) Snapshot of the H3K4me3 landscape at *CDX2* and *OCT4*. The NTU-CAT-derived peaks in nine replicates were visualized using Integrative Genomics Viewer^29^ alongside the peaks from our previous ChIP-seq analysis of a cohort of blastocysts^11^.

The NTU-CAT procedure handled the embryos as cell masses, not as isolated cells; therefore, we validated the penetration of antibodies into embryonic cells by replacing the secondary antibody with a fluorochrome-conjugated one, followed by nuclear counter-staining by Hoechst 33342. The antibodies were confirmed to penetrate the nuclei of most of the embryonic cells (Fig. 3a and Supplementary Movies S1 and S2). In addition, the H3K4me3 profiles at *CDX2* and *OCT4*, which are predominantly expressed in outer cells (trophectoderm) and inner cells (inner cell mass), respectively^13,14^, exhibited similar proportional profiles between ChIP-seq and NTU-CAT (Fig. 3b), further suggesting even penetration of antibodies into the outer and inner cells of the embryos.

**Figure 4:**
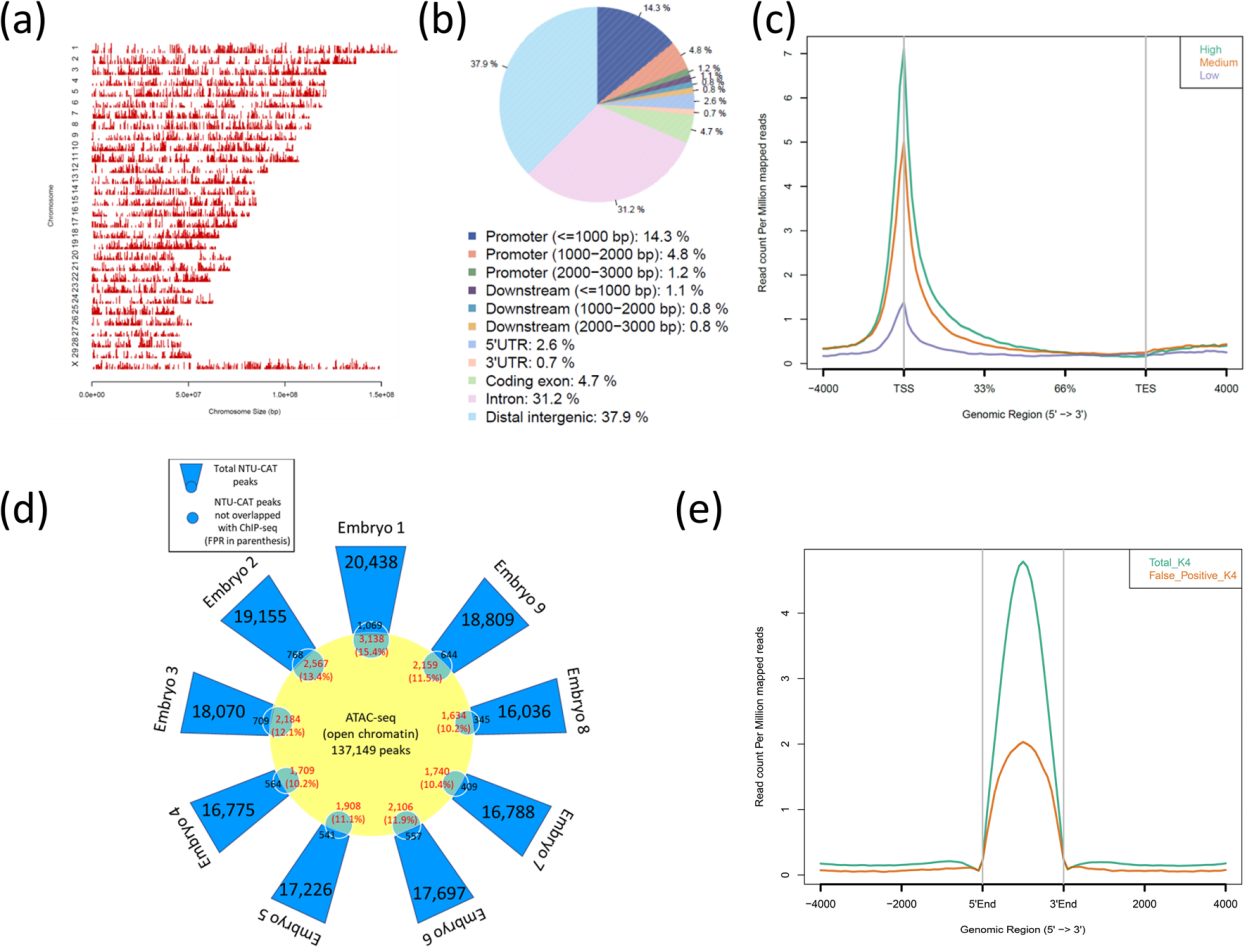
Overview of the NTU-CAT results for H3K4me3 in bovine blastocysts. (**a,b**) Distribution of H3K4me3 peaks in each chromosome (**a**) and in corresponding genic and intergenic regions (**b**). These figures were generated by the CEAS^27^ tool. (**c**) Average profile plot around gene bodies categorized by gene groups with different expression levels based on GSE52415^32^. The figure was generated by ngs.plot^28^. (**d**) The validation of false positive peaks in NTU-CAT possibly due to the open chromatin bias of Tn5 transposase. A girasol plot showing the number of peaks and false positive rate (FPR). The FPR was calculated by the number of NTU-CAT peaks that did not overlap with ChIP-seq (Blastocysts 1 in our previous report^11^) but did overlap with the ATAC-seq peaks^16^, divided by the total number of peaks^15^. (**e**) Average profile plot of the H3K4me3 signals in total NTU-CAT peaks and false positive peaks. The figure was generated by ngs.plot^28^. (**a,b,c,e**) were made using the NTU-CAT Rep1 sample shown in Fig. 2.

We detected approximately 20,000 significant peaks throughout the genome (Fig. 4a) and 20% of the peaks were located on gene promotor regions (Fig. 4b). In addition, average profile plotting around gene bodies categorized by gene groups with different expression levels revealed that highly expressed gene groups had more extensive H3K4me3 modifications (Fig. 4c). Although these results were obtained from single embryos with a smaller number of mapped reads, they are in complete agreement with our previous results using conventional ChIP-seq with a cohort of embryos^11^.

CUT&Tag is a Tn5 transposase-based method^9,10^ and there are concerns of possible bias in epigenomic profiling caused by the preference of Tn5 transposase to cut open chromatin regions^15^. We validated this point in the following analyses. In order to identify the open chromatin regions, we used publicly available ATAC-seq data of bovine inner cell mass (GSM4516360) deposited by Halstead et al.^16^, and mapping and peak calling generated 137,149 ATAC-seq peaks. We then calculated the false positive rate (FPR) possibly caused by the bias of Tn5 transposase toward open chromatin, where the FPR was the number of peaks that did not overlap with ChIP-seq, but did overlap with the ATAC-seq peaks, divided by the total number of peaks, as proposed by Wang et al.^15^. As a result, the FPR was 10–15% (Fig. 4d). Furthermore, the areas of the false positive peaks were relatively smaller than those of all peaks (Fig. 4e).

**Figure 5:**
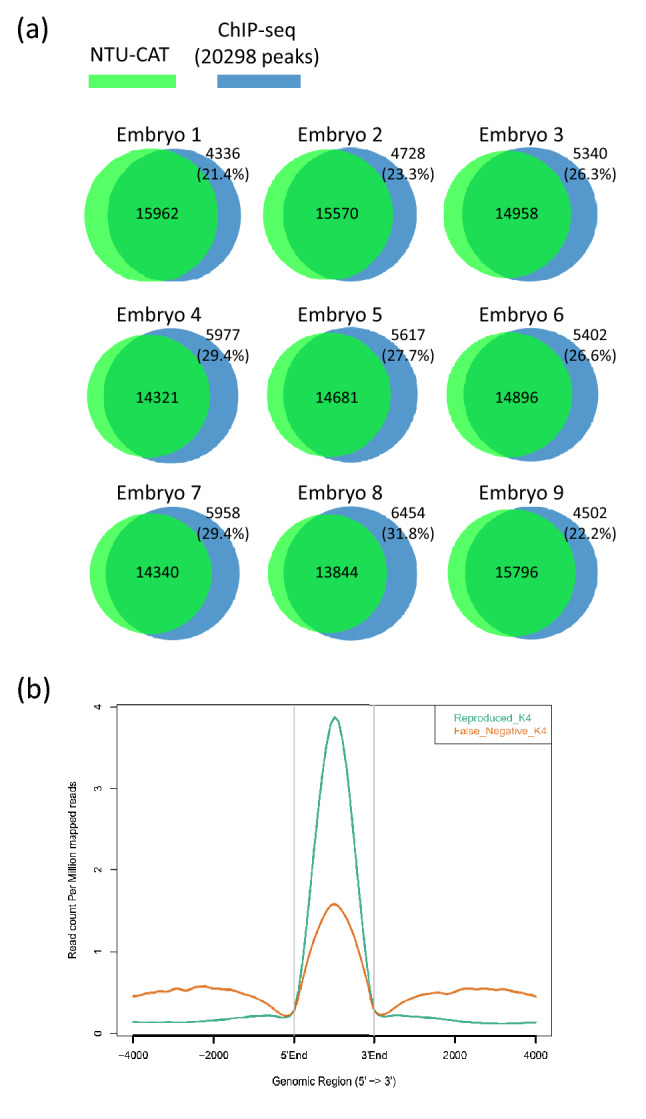
Validation of false negative peaks in NTU-CAT for H3K4me3. (**a**) Calculation of the false negative rate (FNR) in NTU-CAT as the number of H3K4me3 ChIP-seq peaks (the overlapping peaks of Blastocysts 1 and 3 in our previous report^11^), which did not overlap with the respective NTU-CAT peaks divided by the total number of ChIP-seq peaks. (**b**) Average profile plot of the H3K4me3 signals in reproducible and missing (false negative in NTU-CAT) peaks in ChIP-seq compared with NTU-CAT. The overlapping peaks of Blastocysts 1 and 3 in our previous report^11^ were compared with the NTU-CAT Rep1 sample shown in Fig. 2. The figure was generated by ngs.plot^28^ using the Blastocysts 1 profile.

We also calculated the false negative rate (FNR) as the number of H3K4me3 ChIP-seq peaks (the overlapping peaks of Blastocysts 1 and 3 in our previous report^11^) that did not overlap with the respective NTU-CAT peaks divided by the total number of ChIP-seq peaks. The FNR was 21–32% (Fig. 5a). The missing peaks in NTU-CAT had lower signals in ChIP-seq compared with the reproducible peaks (Fig. 5b).

### H3K27me3 profile in blastocysts assessed by NTU-CAT

**Figure 6:**
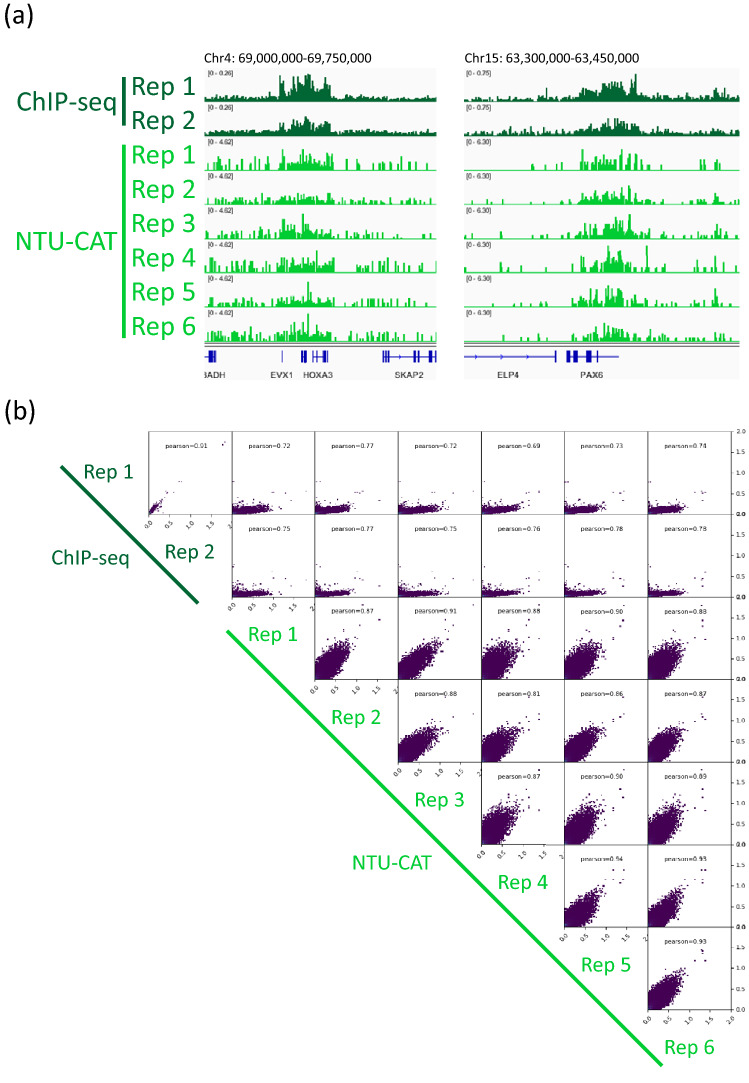
Comparison of the NTU-CAT and ChIP-seq results for H3K27me3 in bovine blastocysts. (**a**) Snapshot of the H3K27me3 landscape in representative positive regions, *HOXA* and *PAX6* genes, respectively. The NTU-CAT-derived peaks in six replicates were visualized using Integrative Genomics Viewer^29^ alongside the peaks from our previous ChIP-seq analysis of a cohort of blastocysts^17^. (**b**) Correlation analysis of duplicates of ChIP-seq and six replicates of NTU-CAT for H3K27me3 in bovine blastocysts. Scatterplots were generated using the same method as in Fig. 2c.

Compared with H3K4me3 modification, H3K27me3 is a typical transcription-repressing marker that generally shows broad distribution^2,4,17^, and we assessed this modification by NTU-CAT. A snapshot of the NTU-CAT peaks from six single blastocysts is shown in Fig. 6a, alongside the peaks from our previous ChIP-seq analysis of a cohort (n =  ~ 15) of blastocysts^17^. The results showed that representative H3K27me3 modifications, such as those on *HOXA* and *PAX6* genes, could be detected using NTU-CAT. We observed typical putative bivalent domains, where H3K4me3 and H3K27me3 coincide^2,4^, and these domains generally enriched the gene ontology (GO) terms that were related to developmental processes (Fig. S1). However, the H3K27me3 peaks obtained by NTU-CAT exhibited a lower resolution compared with those generated by ChIP-seq. In addition, inter- (ChIP-seq vs. NTU-CAT) and intra- (within the same method) assay correlations of the signals were lower compared with the case of H3K4me3 (Fig. 6b).

**Figure 7:**
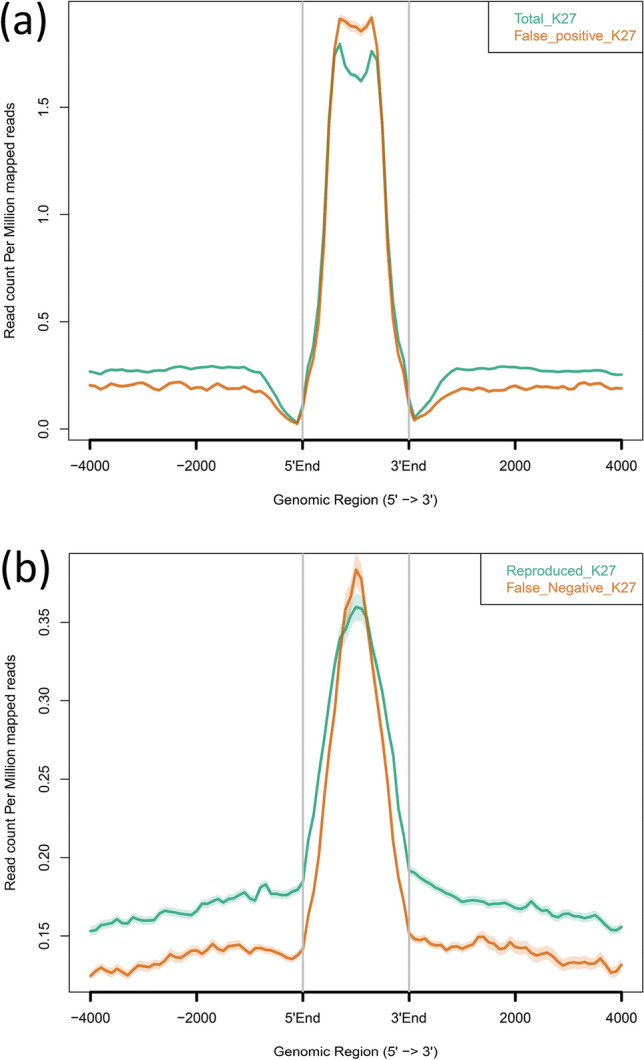
The evaluation of false positive and negative peaks for H3K27me3 in NTU-CAT. (**a**) Average profile plot of the H3K27me3 signals in total NTU-CAT peaks and false positive peaks in the NTU-CAT Rep1 sample shown in Fig. 6a. The figures was generated by ngs.plot^28^. (**b**) Average profile plot of the H3K27me3 signals in reproducible and missing (false negative in NTU-CAT) peaks in ChIP-seq compared with NTU-CAT. The overlapping peaks of H3K27me3_1 and 2 in our previous ChIP-seq report^17^ was compared with the NTU-CAT Rep1 sample shown in Fig. 6a. The figure was generated by ngs.plot^28^ using the H3K27me3_1 profile.

In addition to the relatively lower resolution of H3K27me3 peaks in NTU-CAT, the preference of Tn5 transposase to cut open chromatin regions^15^ could profoundly affect the FPR and FNR. As expected, the FPR (12–25%) was slightly higher than that of H3K4me3 (Table 1). In the calculation of the FNR, a simple intersection of the called peaks was not sufficient because the NTU-CAT H3K27me3 peaks tended to be divided into small pieces compared with the broader ChIP-seq peaks due to the scarcer distribution of the sequence reads. Thus, the ChIP-seq-specific peaks against the original NTU-CAT peaks were filtered again against the NTU-CAT peaks that had been called with the parameter to recognize these pieces as parts of broader peaks (see the “Methods” section). However, even with the correction, the FNR in H3K27me3 was quite high (26–45%, Table 1). Furthermore, in contrast with H3K4me3, the signals of the false positive and negative peaks were comparable to those of the overall peaks (Fig. 7).

**Table 1:** False positive and negative rates (FPR and FNR) in NTU-CAT for H3K27me3.

Replicate	Total peak n	False positive peak n^a^	FPR (%)	False negative peak n	ChIP-seq peak n to be compared^b^	FNR (%)
H3K27me3_Rep1	21,177	5068	23.9	2809	6671	42.1
H3K27me3_Rep2	33,719	5583	16.6	1706	6671	25.6
H3K27me3_Rep3	21,688	5311	24.5	2430	6671	36.4
H3K27me3_Rep4	15,499	1976	12.7	2998	6671	44.9
H3K27me3_Rep5	20,623	2455	11.9	2221	6671	33.3
H3K27me3_Rep6	16,826	2185	13.0	2623	6671	39.3

^a^Compared with merged peaks of GSE171701 of our previous result.

^b^Compared with intersect peaks of GSE171701 of our previous result.

### Typical characterization of single embryos by NTU-CAT – *XIST* activation

**Figure 8:**
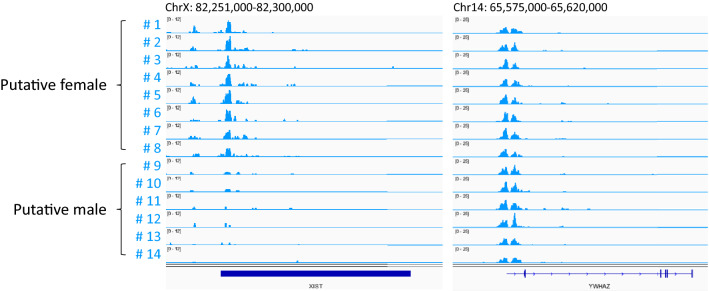
H3K4me3 modifications at the *XIST* locus in each embryo. The right panel shows an example of a gene with sex-independent H3K4me3 modifications, like many others, of *YWHAZ*.

A promising advantage of single embryo analysis is the characterization of individual embryos. To explore the possibility of characterizing individual embryos based on a specific marker that can be detected by NTU-CAT, we focused on H3K4me3 modification of the *XIST* locus. The results showed a clear dichotomy between the presence or absence of H3K4me3 modification at this locus obtained by NTU-CAT (Fig. 8).

## Discussion

In this study, we further modified the CUT&Tag method^9,10^, which can analyze histone modifications in a small number of cells, such that the embryo is handled in the absence of the solid-phase magnetic beads used for antibody and enzyme reactions in the conventional method. The conventional CUT&Tag approach utilizes concanavalin A-coated magnetic beads for fixing dispersed cells to the solid phase in order to facilitate handling and processing of the cells^9,10^. However, preimplantation embryos are cell masses that can be transferred individually to any reaction solution in the experimental process using a fine pipette. In order to more easily handle the embryos in CUT&Tag process, we attempted to use this approach and named the method the “NTU-CAT” (Fig. 1).

As a result, despite some limitations, NTU-CAT generated genome-wide profiles of representative histone modifications, H3K4me3, even from single embryos, comparable to the results of the conventional ChIP-seq method using a cohort of embryos. We demonstrated the overall similarity of the signals detected between ChIP-seq and NTU-CAT (Figs. 2, 3, 4 and 5).

For H3K4me3, some peaks were found to be uniquely more abundant in NTU-CAT (Fig. 2a), and the shape of the average profile of the peaks near the TSSs of genes differed between the two methods such that the “valleys” detected by ChIP-seq were not observed with NTU-CAT (Fig. 2b). This is a general feature of CUT&Tag experiments^9,12^ and may be due to the bias of Tn5 transposase to preferentially cut open chromatin regions^15^. To estimate the magnitude of this bias, we evaluated the generation of false positive peaks by comparing the peaks of NTU-CAT, ChIP-seq^11^, and ATAC-seq^16^. As a result, it was found that there were 10–15% false positive peaks per sample, which were detected possibly due to the open chromatin bias (Fig. 4d). However, the remaining peaks were consistent between ChIP-seq and NTU-CAT, and the average areas of the false positive peaks were relatively smaller than those of the total NTU-CAT peaks (Fig. 4d,e). Furthermore, high correlations of the signals per genomic bins were obtained between ChIP-seq and NTU-CAT (Pearson correlation = 0.87–0.91) and within the NTU-CAT replicates (0.95–0.98, Fig. 2c). Although FNRs were substantial (21–32%, Fig. 5a), this may in part reflect the high background of ChIP-seq compared with NTU-CAT. Supportively, the missing peaks had lower ChIP-seq signals compared with the reproducible peaks (Fig. 5b). However, the substantial FPR and FNR may also reflect the failure of NTU-CAT to detect true peaks due to the Tn5-based bias in terms of chromatin accessibility. This issue is likely to be more serious for the detection of H3K27me3, which is preferentially associated with closed chromatin. The ease of access to the open chromatin region of Tn5 and, conversely, the difficulty of access to the closed chromatin region can result in false positivity and negativity, respectively, for H3K27me3. As expected, the reproducibility between NTU-CAT and ChIP-seq for H3K27me3 (Pearson correlation = 0.69–0.78) was lower compared with that of H3K4me3 (Fig. 6b). Furthermore, the magnitudes of false positive and negative peaks were both comparable to the signals of the overall significant peaks (Fig. 7). A recent systematic benchmarking study of CUT&Tag^18^ also reported low recovery (~ 50%) of ChIP-seq peaks by CUT&Tag in H3K27me3. Collectively, these results and findings suggest a drawback in the evaluation of H3K27me3 peaks in NTU-CAT compared with ChIP-seq and H3K4me3.

Despite this drawback, NTU-CAT was able to detect the typical H3K27me3 peaks that can be detected by ChIP-seq (Fig. 6a and Fig. S1). In addition, the GO analysis of the putative bivalent domains clearly enriched the terms related to developmental processes, consistent with reports that bivalency in embryonic stem cells and blastocysts mark mainly development-related genes^19,20^. Therefore, NTU-CAT, like other CUT&Tag methods, was able to detect representative features of the H3K27me3 modifications. However, we have to acknowledge that the difficulty in evaluating histone modifications in closed chromatin is a drawback of these methods. In addition, true bivalency derived from the same cell states would only be assessable by methods such as re-ChIP^21^ and multimodal CUT&Tag^22^. The low resolution of H3K27me3 peaks in NTU-CAT is in part the result of the scarcer distribution of sequence reads compared with ChIP-seq. Thus, the called peak tended to be divided into small pieces compared with the broader ChIP-seq peaks. Therefore, a more appropriate program for recognizing these pieces as parts of broader peaks may be helpful to overcome this limitation, which we attempted in the filtration of false negative peaks. However, this approach still cannot solve the inherent problem of the Tn5-based bias to open chromatin.

The single embryo-based analysis of histone modifications provides specific details about each embryo. For example, we could detect H3K4me3 modification at the *XIST* locus in about half of the examined embryos, suggesting that these embryos were female and exhibited *XIST* activation leading to X chromosome inactivation (Fig. 8). It is possible that other individual-specific modifications that are detectable by NTU-CAT, such as the *XIST* modification, will be found in the future, and may pave the way for the utilization of epigenetic modifications as diagnostic markers for embryonic characteristics.

Single-cell CUT&Tag has recently been used to profile histone modifications at single-cell resolution^23^. Because this method can be applied to dozens of cells^23^ and also allows for profiling of individual cells, it may have great advantages in characterizing early embryos and is thus worth investigating. Although NTU-CAT cannot capture the characteristics of individual cells, it has the advantage of simplifying the profiling of individual embryos without the labor and equipment required for separating cells, as shown in Fig. 8.

In this study, we used a bovine model because bovine embryos are more similar to human embryos than rodent model in many respects, including mono-ovulatory nature, gamete size, embryonic developmental speed, and the timing of embryonic genome activation, which makes it a clinically important model for the study of human embryos^24^. However, further validation in experimental animal models is needed to explore a wider range of applications for NTU-CAT.

In summary, despite NTU-CAT having some limitations that should be noted, including false positive and negative peaks and lower resolution for broad modifications, we consider that it is a promising replacement for ChIP-seq with the great advantage of being able to analyze individual embryos.

## Methods

### In vitro production of bovine embryos

This study was approved by the Animal Research Committee of Kyoto University (permit numbers R3-10 and R4-10) and was carried out in accordance with the Regulation on Animal Experimentation at Kyoto University. The bovine ovaries used in this study were purchased from a commercial abattoir as by-products of meat processing, and the frozen bull semen used for in vitro fertilization (IVF) was also commercially available. In vitro production of bovine embryos by IVF was performed as previously described^11,17^. Blastocyst-stage embryos at 170 h post IVF (day 7) were collected individually.

### NTU-CAT

The blastocysts were freed from the zona pellucida by using pronase and, from this point on, individual embryos were handled with separate solutions and pipettes to avoid cross-contamination. The basal kit for CUT&Tag was a CUT&Tag-IT Assay Kit (Active Motif). After washing the zona-free blastocysts with phosphate-buffered saline containing 0.01% (w/v) polyvinyl alcohol and 1% (v/v) Protease Inhibitor Cocktail (PIC), they were individually allocated to 50 μL Antibody Buffer with 1 µL (1.4–1.6 µg) of the target primary antibody (C15410003 and C15410069 for H3K4me3 and H3K27me3, respectively, Diagenode), 0.05% (w/v) digitonin, and 1% (v/v) PIC in the wells of a round bottom-shaped 96-well plate. The blastocysts were incubated overnight at 4 °C with gentle shaking (400 rpm). A negative control was set by omitting the primary antibodies. After the primary antibody reaction, the blastocysts were transferred to wells containing 100 µL Dig-Wash buffer with a secondary antibody (guinea pig anti-rabbit IgG antibody), 0.05% (w/v) digitonin, and 1% (v/v) PIC and incubated for 1 h at room temperature (400 rpm). After three washes with 180 µL Dig-Wash Buffer supplemented with digitonin and PIC in the 96-well plate, the blastocysts were transferred to wells containing 100 µL Dig-300 Buffer with pA-Tn5 transposomes, 0.01% (w/v) digitonin, and 1% (v/v) PIC and incubated for 1 h at room temperature (400 rpm). After three washes with 180 µL Dig-300 Buffer supplemented with digitonin and PIC on the 96-well plate, the blastocysts were transferred to individual microcentrifuge tubes (Eppendorf 0030 108.051) containing 125 µL Tagmentation Buffer with 0.01% (w/v) digitonin and 1% (v/v) PIC and incubated for 1 h at 37 °C without shaking.

After tagmentation, 4.2 μL of 0.5 M EDTA, 1.25 μL of 10% SDS and 1.1 μL of 10 mg/mL proteinase K were added to the tubes and incubated for 1 h at 55 °C or overnight at 37 °C with vigorous shaking (1,300 rpm). The tubes were heated at 70 °C for 20 min to inactivate proteinase K, and then cooled to room temperature. SPRIselect beads (Beckman Coulter, 145 µL) were added to the tubes, vortexed for 1 min, and allowed to incubate for 10 min. The tubes were placed on a magnet stand for 4 min to collect the magnetic beads and the liquid was removed. The beads were washed twice with 1 mL of 80% ethanol. After drying the bead pellets for 2–5 min, 35 µL DNA Purification Elution Buffer was added and the tubes were vortexed and left to stand for 5 min. The tubes were placed on a magnet stand for 4 min to collect the magnetic beads and the liquid containing tagmented DNA was transferred to PCR tubes.

PCR amplification of sequencing libraries was performed in a volume of 50 µL using 30 µL tagmented DNA and i7 and i5 indexing primers according to the manufacturer’s protocol. The PCR condition was as follows: 72 °C for 5 min; 98 °C for 30 s; 20 cycles of 98 °C for 10 s and 63 °C for 10 s; final extension at 72 °C for 1 min; and hold at 10 °C. Post-PCR library purification was performed with 55 µL SPRIselect beads (vortex 1 min, stand for 5 min, and bead collection 4 min) and 180 µL of 80% ethanol as described above. Finally, the sequencing libraries were eluted in 25 µL DNA Purification Elution Buffer.

### Assessment of antibody permeability in NTU-CAT

The permeability of the antibodies in NTU-CAT procedure was assessed by replacing the secondary antibody with Alexa Fluor 488-conjugated goat anti-rabbit IgG (A11008; Thermo Fisher Scientific). The nuclei of blastocysts were counterstained with 10 μg/mL Hoechst 33342 in phosphate-buffered saline containing 0.05% (v/v) Tween 20 (PBST) for 20 min. The samples were then washed twice with PBST, mounted onto slides with a droplet of Vectashield mounting medium (Vector Laboratories), and flattened by the dead weight of the coverslip. The slides were examined under a fluorescence microscope (BX50; Olympus) equipped with a WU filter for Hoechst and a WIB filter for Alexa Fluor 488. In addition, one-side flattened samples (blastocysts) were prepared by maintaining the distance from the coverslip on the slides and then examined under a confocal laser scanning microscope (SP8 FALCON; Leica Microsystems). The confocal images were acquired as 12-µm thick Z-stacks at 0.7-µm intervals.

### DNA sequencing and data processing

Sequencing was performed on a HiSeqX (Illumina) as paired-end 150-base reads. The sequencing reads were quality-checked, merged, and aligned to the bovine genome (Bos_taurus_UMD_3.1.1/bosTau8, June 2014) using Bowtie 2^25^. Handling of sam and bam files was performed by Samtools (http://www.htslib.org/). Mapping duplicates were removed by Picard (http://broadinstitute.github.io/picard/). The generated bam files were converted to bigWig (bw) files by the bamCoverage tool of deepTools (https://deeptools.readthedocs.io/en/develop/) with counts-per-million normalization. The correlation plots between the experiments were made from the bw files fed to deepTools with the command “multiBigwigSummary bins --bwfiles [bw file names] -o [output name].npz,” followed by the command “plotCorrelation -in [npz file name] -c pearson -p scatterplot -o [output name] --removeOutliers --plotFileFormat png --xRange [X axis range] --yRange [Y axis range] --log1p.” The H3K4me3 peaks were called using MACS1.4^26^ with the following command: “macs14 -t [bam file name] --name = [output file name] -f BAM -g 2.7e9 -S --wig.” The H3K27me3 peaks were called with following options: “--nomodel --nolambda --shiftsize = 300.” The annotation of called peaks to genomic regions was generated using CEAS^27^ with the following command: “ceas --name = [output file name] --bg -g bosTau8.db -b [bed file name] -w [wig file name].” Average H3K4me3 signal profiles were generated by ngs.plot^28^ with the following command: “ngs.plot.r -G bosTau6 -R [region name] -C [txt file name of configuration] -O [output file name] -L 4000 -FL 200.” Peaks were visualized using Integrative Genomics Viewer^29^. The common and specific peaks between samples were identified using bedtools (https://bedtools.readthedocs.io/en/latest/) with the default and -v option, respectively, and these were used to calculate the FPR, FNR, and putative bivalent domains. In the calculation of H3K27me3 FNR, the ChIP-seq-specific peaks against NTU-CAT peaks were filtered again against the H3K27me3 peaks that had been called with the --shiftsize = 2400 option in order to avoid overestimation of the false negative peaks due to the splitting of broad peaks in NTU-CAT. GO analysis was performed using the DAVID tool with the GO_Fat categories for biological processes^30,31^.

### Publicly available data

The following raw data from publicly available databases were used: ChIP-seq of bovine blastocysts from our previous studies^11,17^, i.e., rep1 and rep3 of GSE161221 (H3K4me3) and rep1 and rep2 of GSE171701 (H3K27me3); RNA-seq of bovine blastocysts, Blastocyst_replicate1, 2, and 3 of GSE52415^32^; and ATAC-seq of bovine inner cell mass, ICM_rep1 of GSE143658^16^. For RNA-seq data, the three (blastocyst) datasets were merged and expression levels in RPKM values were calculated as previously described^33^. The genes were evenly divided into three categories as high, medium, and low expression levels according to the calculated RPKM values.

## Supplementary Information

Supplementary Video 1.

Supplementary Video 2.

Supplementary Information.

## Data Availability

The NTU-CAT processed data have been deposited at Zenodo (https://zenodo.org/record/6002122). The raw sequence data are available upon request; please contact us at the following address: ikeda.syuntaro.6u@kyoto-u.ac.jp.
