# Supplementary material for: Genome-wide profiling of histone H3K4me3 and H3K27me3 modifications in individual blastocysts by CUT&Tag without a solid support (NON-TiE-UP CUT&Tag)
Source: Sci Rep. 2022 Jul 11;12:11727. doi: 10.1038/s41598-022-15417-x (PMC9276795; doi:10.1038/s41598-022-15417-x)
Supplement: Supplementary file 3 — Supplementary Information. [file 41598_2022_15417_MOESM3_ESM.pdf]

Supplementary Information for

Genome-wide profiling of histone H3K4me3 and H3K27me3 modifications in individual blastocysts by CUT&Tag without a solid support (NON-TiE-UP CUT&Tag)

Kazuki Susami, Shuntaro Ikeda*, Yoichiro Hoshino, Shinnosuke Honda, Naojiro Minami

Laboratory of Reproductive Biology, Graduate School of Agriculture, Kyoto University, Kyoto 606-8502, Japan

*Corresponding author

Email: ikeda.syuntaro.6u@kyoto-u.ac.jp

https://orcid.org/0000-0002-4939-2135

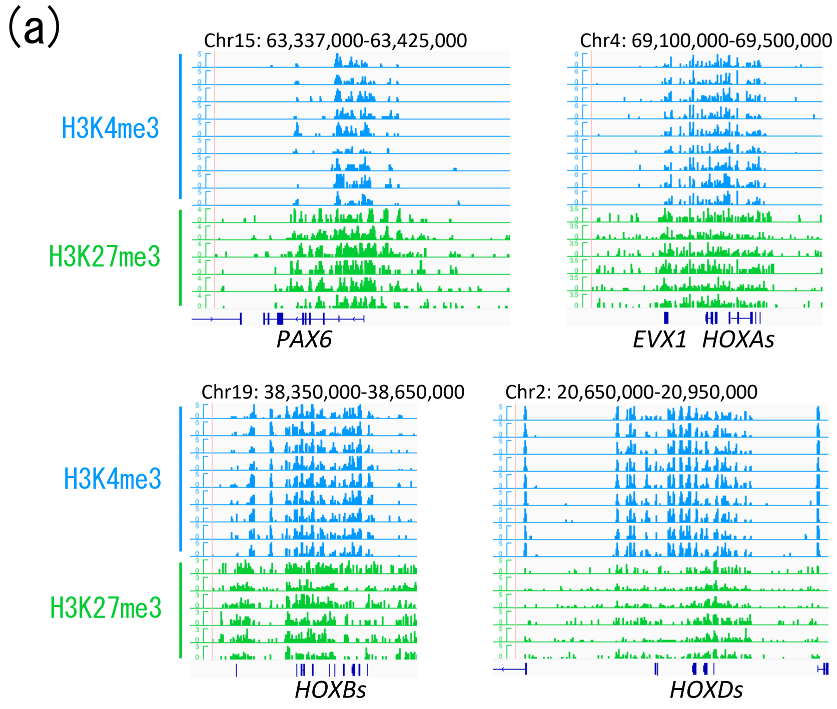

(b)

H3K4me3_Rep1

Top 10 GO term	Gene n.	FDR
regulation of nucleobase-containing compound metabolic process	102	4.1E-08
positive regulation of nitrogen compound metabolic process	58	1E-07
regulation of RNA metabolic process	93	2.4E-07
positive regulation of nucleobase-containing compound metabolic process	54	2.4E-07
regulation of gene expression	105	3.3E-07
positive regulation of cellular metabolic process	79	4.8E-07
negative regulation of macromolecule biosynthetic process	46	0.000001
negative regulation of gene expression	51	1.3E-06
positive regulation of cellular biosynthetic process	52	1.3E-06
negative regulation of macromolecule metabolic process	65	1.3E-06

Bivalent domain with H3K27me3_Rep1

regulation of transcription from RNA polymerase II promoter	68	0.00035
transcription, DNA-templated	81	0.00053
negative regulation of developmental process	32	0.0012
tube development	25	0.0012
embryo development	35	0.0012
regulation of RNA metabolic process	83	0.0013
sensory organ development	24	0.0013
nervous system development	54	0.0015
nucleic acid-templated transcription	81	0.0015
camera-type eye development	17	0.0015

Bivalent domain with H3K27me3_Rep2

nervous system development	68	8.3E-10
neuron differentiation	50	8.3E-10
regulation of transcription from RNA polymerase II promoter	77	1.1E-09
epithelium development	45	1.5E-09
neurogenesis	55	1.5E-09
animal organ morphogenesis	43	1.9E-09
generation of neurons	52	2.5E-09
regulation of cell differentiation	56	2.9E-09
cell development	64	2.9E-09
embryo development	43	8.4E-09

Bivalent domain with H3K27me3_Rep3

regulation of transcription from RNA polymerase II promoter	79	4.2E-10
epithelium development	42	2.4E-07
embryo development	41	4.2E-07
transcription from RNA polymerase II promoter	53	5.8E-07
transcription, DNA-templated	84	0.000001
negative regulation of developmental process	36	1.2E-06
embryonic morphogenesis	29	1.2E-06
animal organ morphogenesis	38	1.2E-06
regulation of RNA metabolic process	88	1.2E-06
regulation of nucleic acid-templated transcription	84	1.5E-06

Figure S1. Putative bivalent domain in NTU-CAT. (a) Representative putative bivalent domains observed in the NTU-CAT experiments. The NTU-CAT landscape of H3K4me3 (n = 9) and H3K27me3 (n = 6) at these loci are shown. (b) The top 10 significant GO terms for biological processes enriched by the genes with the top 300 highest peak occupancy rates at TSSs in NTU-CAT. The table on the left shows the result from the H3K4me3 peaks in Rep1 of H3K4me3. The tables on the right show the results from the bivalent peaks of the sample generated from the comparison with the Rep1–3 samples of H3K27me3 in NTU-CAT. "Gene n." and "FDR" indicate the numbers of related genes and the false discovery rate, respectively.

Legends of Supplementary Movies

Movie S1. Permeability of the H3K4me3 antibodies in NTU-CAT assessed by a confocal laser scanning microscope. Twelve-micrometer Z-stack image movie that cross from the free side to the flattened side of the representative sample was made by stacking 17 confocal images at 0.7-μm intervals. H3K4me3 (green) and Hoechst nuclear counter-staining (blue) are shown on the left and right, respectively.

Movie S2. Permeability of the H3K27me3 antibodies in NTU-CAT assessed by a confocal laser scanning microscope. Twelve-micrometer Z-stack image movie that cross from the free side to the flattened side of the representative sample was made by stacking 17 confocal images at 0.7-μm intervals. H3K27me3 (green) and Hoechst nuclear counter-staining (blue) are shown on the left and right, respectively.

Supplementary table

Table S1. Data generated by NTU-CAT of bovine blastocysts in this study				
Replicate	Total reads	Total mapped reads (%) [1]	Uniqulely mapped reads (%)	Reads after de-duplication [2]
H3K4me3_Rep1	309,069,780	290,859,627 (94.11)	253,440,564 (82.00)	1,517,565
H3K4me3_Rep2	271,397,942	254,467,978 (93.76)	226,414,835 (83.43)	1,366,333
H3K4me3_Rep3	256,869,432	240,626,890 (93.68)	210,370,289 (81.90)	1,028,471
H3K4me3_Rep4	183,987,624	172,417,072 (93.71)	152,025,702 (82.63)	936,256
H3K4me3_Rep5	188,117,734	175,622,708 (93.36)	155,765,943 (82.80)	978,502
H3K4me3_Rep6	191,796,858	180,940,019 (94.34)	159,421,116 (83.12)	1,153,830
H3K4me3_Rep7	182,234,060	172,129,980 (94.46)	150,799,519 (82.75)	886,026
H3K4me3_Rep8	137,009,656	128,671,827 (93.91)	109,848,249 (80.18)	798,472
H3K4me3_Rep9	172,778,388	161,698,547 (93.59)	143,109,357 (82.83)	1,258,822
H3K27me3_Rep1	194,624,022	183,087,772 (94.07)	126,286,155 (64.89)	845,622
H3K27me3_Rep2	196,073,114	184,942,697 (94.32)	131,068,624 (66.85)	1,555,708
H3K27me3_Rep3	177,644,032	167,949,176 (94.54)	123,051,701 (69.27)	1,173,395
H3K27me3_Rep4	294,872,164	281,759,640 (95.55)	194,676,393 (66.02)	793,021
H3K27me3_Rep5	187,539,216	178,467,948 (95.16)	128,827,707 (68.69)	1,176,819
H3K27me3_Rep6	371,009,066	353,574,975 (95.3)	260,924,371 (70.33)	1,081,875
[1] Mapped using Bowtie 2.				
[2] Processed by Samtools and Picard.				
